# Engineering the acid-tolerant yeast *Issatchenkia orientalis* for efficient lactic acid production via optimized glucose and xylose utilization

**DOI:** 10.3389/fmicb.2026.1951033

**Published:** 2026-09-11

**Authors:** Yejin Lin, Ayoung Kim, Daeun Lee, Nam Kyu Kang

**Affiliations:** Department of Chemical Engineering, College of Engineering, Kyung Hee University, Yongin-si, Gyeonggi-do, Republic of Korea

**Keywords:** carbon catabolite repression, *Issatchenkia orientalis*, lactic acid, mixed-sugar co-utilization, sugar transporters

## Abstract

The acid-tolerant yeast *Issatchenkia orientalis* is a promising platform for the sustainable production of organic acids. However, the inefficient conversion of lignocellulosic biomass-derived sugars, primarily due to carbon catabolite repression (CCR), reduces overall production efficiency and limits its industrial application. In this study, we established a targeted genetic framework for efficient glucose–xylose co-utilization by coordinating hexokinase (HXK) modulation and transport-level engineering. Sequential fed-batch fermentations revealed that a xylose-initiated feeding strategy achieved a 2.04-fold higher lactic acid yield than simultaneous fermentation. To bypass carbon catabolite repression, endogenous hexokinases were characterized, and single deletions (*hxk*1Δ, *hxk*2Δ, or *hxk*3Δ) were conducted to attenuate glucose dominance. While this approach improved lactic acid yields, it simultaneously imposed severe kinetic bottlenecks. To address these limitations, heterologous sugar transporters, plant-derived *AtSWEET7* and yeast-derived *LST1*, were integrated. Characterization in the intact background revealed that the Major Facilitator Superfamily (MFS)-type *LST1* from *Lipomyces starkeyi* outperformed *AtSWEET7*. Double-copy integration of *LST1* yielded the engineered SD108XL-LST2 strain, which achieved a lactic acid titer of 53.4 g/L within 56 h from a mixed-sugar substrate containing approximately 45 g/L glucose and 44 g/L xylose. Notably, the final yield (0.63 g/g) and volumetric productivity (0.96 g/L·h) represented 57.5% and 47.7% increases over the parental SD108XL strain, respectively. This transport-driven strategy effectively overrides native metabolic hierarchies while preserving essential glycolytic signaling, offering a robust framework for high-efficiency lignocellulosic biorefineries for organic acid production.

## 1 Introduction

*Issatchenkia orientalis* (syn. *Pichia kudriavzevii*) has emerged as a highly promising non-conventional yeast platform owing to its remarkable tolerance to extremely acidic conditions and innate resilience to a wide range of lignocellulose-derived inhibitors, including acetic acid and furfural (Park et al., 2018; Seong et al., 2017). Unlike the traditional model yeast, *Saccharomyces cerevisiae*, which is Crabtree-positive and tends to divert carbon flux toward ethanol even under aerobic conditions, *I. orientalis* is generally Crabtree-negative, allowing it to maintain a high metabolic flux and robust biomass growth even at high glucose concentrations (Imura et al., 2020; Xiao et al., 2022). These unique physiological advantages position *I. orientalis* as an ideal microbial factory for the sustainable production of organic acids from lignocellulosic biomass-derived sugars directly in their free-acid form, thereby bypassing the costly and waste-intensive neutralization steps (Suthers and Maranas, 2022; Xi et al., 2023; Tan et al., 2025).

Lignocellulosic biomass, comprising agricultural residues, forestry waste, and non-edible energy crops, represents the most abundant and sustainable carbon source for the transition toward a bio-based economy (Segers et al., 2024; Moustafa et al., 2026). Upon efficient pretreatment and enzymatic hydrolysis, this complex biomass releases a diverse mixture of monosaccharides, primarily consisting of 40%−50% cellulose-derived glucose and 20%−30% hemicellulose-derived xylose (Wang et al., 2025; Mosier et al., 2005). For a biorefinery to be economically viable, the near-complete conversion of both sugar fractions is essential for maximizing the carbon yield and minimizing production costs (Kim et al., 2013; Eiteman et al., 2008). However, the presence of a robust carbon catabolite repression (CCR) mechanism in most industrial microorganisms remains a major technical obstacle (Gancedo, 1998; Kuyper et al., 2005). Even when the heterologous xylose catabolic pathway, comprising xylose reductase (XR), xylitol dehydrogenase (XDH), and xylulokinase (XK), is successfully introduced to enable pentose assimilation, cellular preference for glucose triggers a strict regulatory hierarchy (Jeffries, 2006; Hapeta et al., 2021). This hierarchy leads to the sequential utilization of sugars, where xylose consumption is repressed or delayed until the glucose is entirely exhausted, resulting in prolonged fermentation cycles, metabolic decoupling, and significantly reduced overall productivity in mixed-sugar environments (Jeong et al., 2020; Goncalves et al., 2014).

To overcome these metabolic constraints and achieve efficient glucose–xylose utilization, various engineering strategies have been developed that focus on metabolic rewiring and transport optimization (Hou et al., 2017; Reider Apel et al., 2016). One prominent approach involves constructing xylose-dedicated strains by deleting the primary hexokinase (HXK) genes, thereby blocking glucose phosphorylation and forcing cells to rely on xylose as the sole carbon source (Muller et al., 2022; Bae et al., 2014; Shin et al., 2024). However, several studies have reported that these engineering approaches often face significant kinetic challenges, in which the absence of glucose-derived signals prevents the induction of the necessary transport and catabolic machinery for alternative sugars (Moriya and Johnston, 2004; Gancedo, 1998). This suggests that metabolic rewiring at the phosphorylation level is insufficient if xylose entry remains restricted by the native transport system, which typically exhibits low affinity for pentoses and is highly sensitive to glucose inhibition (Nijland and Driessen, 2020; Goncalves et al., 2014). Consequently, transport-level engineering has attracted considerable interest as a more direct and effective strategy to bypass native regulatory bottlenecks (Nijland and Driessen, 2020; Farwick et al., 2014). Consequently, transport-level engineering has attracted considerable interest as a more direct and effective strategy to mitigate native regulatory bottlenecks (Nijland and Driessen, 2020; Farwick et al., 2014). Furthermore, recent studies have increasingly emphasized that broader transporter engineering strategies, ranging from the rational mutagenesis of native sugar permeases to the bioprospecting of novel heterologous transporters, serve as powerful tools to effectively circumvent carbon catabolite repression and enable simultaneous mixed-sugar utilization across diverse microbial platforms (Taveira et al., 2024; Donzella et al., 2026; Kuanyshev et al., 2026). Specifically, the heterologous expression of transporters such as LST1 (from *Lipomyces starkeyi*) and AtSWEET7 (from *Arabidopsis thaliana*) has been identified as a breakthrough, as these transporters can facilitate robust xylose influx in *S. cerevisiae* independently of extracellular glucose concentrations (Kuanyshev et al., 2021; Taveira et al., 2024). By strengthening the pull of xylose catabolism through transporter-level decoupling, it becomes possible to achieve efficient utilization of glucose and xylose without the pleiotropic growth defects often observed in global transcriptional engineering (Nijland et al., 2014; Lee et al., 2015).

In this study, we engineered *I. orientalis* to maximize its potential for lignocellulosic sugar conversion by coordinating hexokinase modulation and transporter optimization. We investigated the functional roles of individual hexokinase genes (*HXK1, HXK2*, and *HXK3*) and identified their contributions to the glucose catabolic hierarchy. To circumvent the resulting metabolic trade-offs, we integrated heterologous transporters, focusing on gene-dosage optimization of *LST1* from *Lipomyces starkeyi* to enhance the pull of xylose catabolism. The novelty of this work lies in demonstrating, for the first time, that such targeted transport-level decoupling can effectively override the strict carbon catabolite repression inherent in *I. orientalis*, thereby enhancing the production of organic acids, especially lactic acid, under mixed-sugar conditions. This work establishes a targeted genetic framework for developing high-performance yeast strains that efficiently and concurrently utilize mixed sugars for sustainable organic acid production.

## 2 Materials and methods

### 2.1 Cultivation conditions for *Issatchenkia orientalis*

The *I. orientalis* strains constructed and utilized in this study are summarized in Table 1. For the preparation of seed cultures, cells were grown overnight (approximately 16 h) in 5 ml of YPD20 medium [1% (w/v) yeast extract, 2% (w/v) peptone, and 20 g/L glucose] at 30 °C with shaking at 250 rpm. Subsequently, the pre-cultured cells were inoculated into 25 mL of YP medium supplemented with glucose or xylose, and the initial optical density (OD_600_) was adjusted to 1.0. The exact initial concentrations of glucose and xylose in the mixed-sugar medium varied slightly depending on the specific experiment; thus, approximate concentrations are mentioned in the text. For lignocellulosic fermentation, a YP-rice straw medium was prepared. Briefly, 10 wt% dried rice straw was pretreated with 2 wt% sulfuric acid at 121 °C for 60 min, neutralized to pH 5.0–5.5, and enzymatically hydrolyzed using 4 wt% Cellic^®^ CTec3 at 50 °C for 72 h. The clarified supernatant was supplemented with 1% yeast extract and 2% peptone, followed by sterile filtration. These main cultivations were conducted in 125-ml Erlenmeyer flasks with a 25 ml working volume at 30 °C. Aeration was maintained by agitation at 250 rpm. To stabilize the pH during fermentation, calcium carbonate (CaCO_3_) was added at a final concentration of 50 g/L as a buffering agent. Fermentations were continued until the supplied carbon sources were nearly depleted or until sugar consumption completely ceased. All fermentation experiments were performed in biological duplicates. Data are presented as the mean ± standard deviation. All individual raw data points from the replicates are provided in the Supplementary Materials.

**Table 1:** Strains used in this study.

Strain	Description	Source
SD108WT	*Issatchenkia orientalis* wild-type	(Lee et al., 2022)
SD108XL	SD108/*pdc*Δ/*gpd*Δ/*ura3*Δ/IS1::LDH/IS3::XR-XDH-XK	(Lee et al., 2024)
SD108XL/*hxk1*Δ	SD108XL with *HXK1* deletion	This study
SD108XL/*hxk2*Δ	SD108XL with *HXK2* deletion	This study
SD108XL/*hxk3*Δ	SD108XL with *HXK3* deletion	This study
SD108XL/*hxk123*Δ	SD108XL with *HXK1, HXK2*, and *HXK3* deletion	This study
SD108XL/*hxk123*Δ/LST	SD108XL/*hxk*123Δ with LST1 integrated at the IS5 locus	This study
SD108XL/*hxk123*Δ/AS	SD108XL/*hxk*123Δ with *AtSWEET7* integrated at the IS5 locus	This study
SD108XL-AS	SD108XL with *AtSWEET7* integrated at the IS5 locus	This study
SD108XL-LST1	SD108XL with LST1 integrated at the IS5 locus	This study
SD108XL-LST2	SD108XL-LST1 with LST1 integrated at the IS2 locus	This study

### 2.2 Plasmid construction

All plasmids, genes, and primers used in this study are summarized in Supplementary Tables S1–S3, respectively. *Escherichia coli* DH5α was used as the host for plasmid cloning and propagation. Cells were cultured at 37 °C in Luria-Bertani (LB) medium supplemented with appropriate antibiotics (100 μg/ml ampicillin or 50 μg/ml kanamycin) for selection. The heterologous *AtSWEET7* and *LST1* genes were synthesized (Supplementary Table S2). In contrast, donor DNA fragments for *HXK* disruption and genomic integration cassettes were amplified via PCR using specific primers (Supplementary Table S3) and Phusion High-Fidelity DNA Polymerase (New England Biolabs, USA). Expression vectors, pTOP V2_AS and pTOP V2_LST (Supplementary Table S1), were constructed using the pTOP V2 backbone (Enzynomics, Republic of Korea). The synthesized genes were codon-optimized for *I. orientalis* and integrated into the vector along with the endogenous P_TDH3_ promoter and T_MDH1_ terminator via Gibson Assembly (New England Biolabs, USA). For genome editing, the pCast vector containing the Cas9 and gRNA expression cassettes was utilized for CRISPR/Cas9-mediated engineering. The vector backbone was linearized with AarI (Enzynomics, Republic of Korea), and guide RNA (gRNA) cassettes targeting *HXK1, HXK2, HXK3*, IS2, and IS5 were produced using the primers listed in Supplementary Table S2 and assembled using T4 DNA ligase (New England Biolabs, USA). The previously identified integration sites IS2 (GenBank: JQFK01000382, coordinates 629–659) and IS5 (GenBank: JQFK01000002, coordinates 280,647–280,669) were utilized (Lee et al., 2022, 2024).

### 2.3 Strain construction

*I. orientalis* cells were transformed using a modified lithium acetate (LiAc)-based chemical procedure (Lee et al., 2024). Briefly, cells were grown overnight in 5 ml of YPD medium and used to inoculate 25 ml of fresh YPD broth until reaching an OD600 of approximately 0.8. The harvested cells were washed twice with sterile distilled water and prepared for transformation. The transformation mixture consisted of 240 μl of 50% (w/v) PEG 3350, 36 μL of 1 M LiAc, 10 μl of denatured salmon testes carrier DNA (10 mg/ml). For CRISPR/Cas9-mediated genome editing, approximately 1 μg of the pCast plasmid (expressing Cas9 and the target-specific guide RNA) and 1.5 μg of the corresponding repair DNA fragment were co-transformed into the cells (Supplementary Tables S1, S2). After incubation at 42 °C for 60 min, cells were recovered in 700 μl of 2X YP medium at 30 °C for 4 h with agitation to allow for phenotypic expression. The recovery culture was concentrated and spread onto appropriate selective agar plates containing 120 μg/ml nourseothricin (cloNAT), where transformants were obtained after 3–4 days of incubation at 30 °C. For sequential genetic modifications, the pCast plasmid was cured to recycle the selectable marker by transferring the cells more than five times in an antibiotic-free YP medium containing 20 g/L glucose. To confirm successful genomic integration or gene disruption, colony PCR was performed using diagnostic primers (Supplementary Table S3). Cell lysates were prepared by suspending a colony in 0.2 M NaOH, then thermally lysing and centrifuging to isolate the template DNA. PCR amplification was carried out using DreamTaq DNA Polymerase (Thermo Fisher Scientific, USA) to verify specific genetic modifications. For long-term storage, all engineered *I. orientalis* strains were preserved as 20% (v/v) glycerol stocks and stored at −80 °C.

### 2.4 Analytical methods

Cell growth was monitored by measuring the optical density at 600 nm (OD_600_) using a UV/Vis spectrophotometer (Nabi, MicroDigital Co., Ltd., Seoul, Republic of Korea). OD_600_ was determined after dilution with 0.2 M HCl to eliminate turbidity interference from residual CaCO_3_. For metabolite analysis, culture samples were centrifuged at 15,928 × g for 10 min to remove cells, and the resulting supernatants were used to quantify extracellular sugars and organic acids. Concentrations of glucose, xylose, and lactic acid products were determined by high-performance liquid chromatography (HPLC, Agilent 1260 Series; Agilent Technologies, Wilmington, NC USA) equipped with a refractive index detector and a Rezex ROA-Organic Acid H^+^ (8%) ion-exclusion column (Phenomenex Inc., Torrance, CA, USA) The separation was performed at 50 °C using 0.005 M H_2_SO_4_ as the mobile phase at a flow rate of 0.5 ml/min, with an injection volume of 20 μl. Calibration curves were generated using four-point external standards (0.5, 1, 2.5, and 5 g/L) prepared from analytical-grade glucose, xylose, and lactic acid (Sigma-Aldrich, St. Louis, MO, USA). The linearity of the calibration curves was highly conserved (*R*^2^ > 0.99) within the analyzed concentration ranges. The limit of detection was estimated to be approximately 0.05 g/L for the quantified metabolites. To quantitatively evaluate fermentation performance, the total lactic acid yield and productivity were calculated as follows:


$$\begin{array}{l} Yield\;\left(g/g\right)\;=\;\frac{\;Lactic\;acid\;concentration\;\left(g/L\right)}{Concentration\;of\;consumed\;carbon\;sources\;\left(g/L\right)} \end{array}$$

$$\begin{array}{l} Productivity\;\left(g/L\right)=\;\frac{\;Lactic\;acid\;concentration\;\left(g/L\right)}{Fermentation\;time\;\left(h\right)} \end{array}$$

## 3 Results and discussion

### 3.1 Effect of sugar feeding order on glucose–xylose co-utilization for lactic acid production

**Figure 1:**
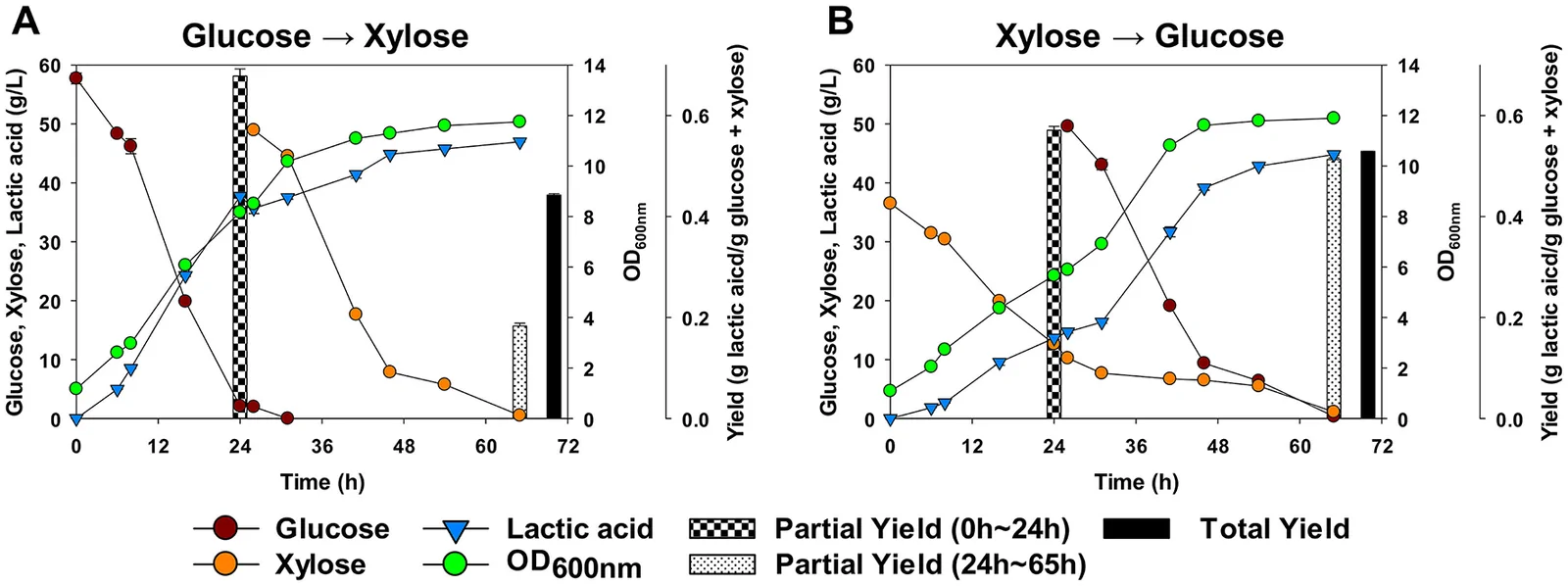
Impact of sugar feeding strategies on the mixed-sugar fermentation profiles of engineered *I. orientalis* SD108XL. **(A)** Fermentation profile under the glucose-initiated feeding strategy. Glucose was initially provided at 57.7 g/L, followed by the addition of 49.6 g/L xylose at 26 h. **(B)** Fermentation profile under the xylose-initiated feeding strategy. Xylose was initially provided at 36.5 g/L, followed by the addition of 49.0 g/L glucose at 24 h. Fermentations were conducted in YP medium (25 ml in 125-ml Erlenmeyer flasks) at 30 °C with shaking at 250 rpm. To control the pH during fermentation, CaCO_3_ was added at a concentration of 50 g/L as a buffering agent. Data points and error bars represent the mean ± standard deviation from biological duplicates.

In our previous study, the engineered *I. orientalis* strain SD108XL demonstrated efficient D-lactic acid production via the expression of a heterologous D-lactate dehydrogenase gene from *Leuconostoc mesenteroides*; however, it was limited by native carbon catabolite repression (CCR), resulting in a low total lactic acid yield of 0.26 g/g (calculated as g of lactic acid produced per g of total sugars consumed) under simultaneous glucose–xylose fermentation conditions. To investigate the impact of the chronological order of substrate exposure on metabolic efficiency, we performed sequential fed-batch fermentations (Figure 1). Under the glucose-initiated feeding strategy (Figure 1A), the strain consumed almost all of the available glucose (57.7 g/L) within the first 26 h, producing 35.7 g/L of lactic acid. However, upon subsequent xylose feeding, metabolic activity slowed. Indeed, 49.6 g/L of xylose was consumed over the next 39 h, yielding only 11.3 g/L of lactic acid. This resulted in a total lactic acid yield of 0.44 g/g (based on total consumed sugar), with a specific xylose-to-lactic acid yield of only 0.18 g/g (based solely on the consumed xylose). In contrast, the xylose-initiated feeding strategy exhibited a markedly different profile. Under this strategy, the strain consumed 26.3 g/L of the initially provided 36.0 g/L xylose within the first 26 h, producing 14.7 g/L of lactic acid (Figure 1B). Upon the subsequent addition of 49.0 g/L of glucose, the strain rapidly co-utilized the remaining xylose and the newly added glucose to produce an additional 30.1 g/L of lactic acid, resulting in a total lactic acid yield of 0.53 g/g (based on total consumed sugars). Compared to simultaneous fermentation (0.26 g/g), these results represent a 1.7-fold higher yield for the glucose-first strategy (0.44 g/g) and a 2.04-fold higher yield for the xylose-first strategy (0.53 g/g). Remarkably, the specific xylose-to-lactic acid yield in the xylose-initiated conditions reached 0.52 g/g (calculated as the ratio of lactic acid produced to xylose consumed during the initial phase), which is approximately 2.8-fold higher than that of the glucose-initiated strategy.

These results suggest that the metabolic state established during the initial phase of fermentation dictates the efficiency of subsequent pentose utilization. We hypothesize that the lower xylose conversion efficiency observed in the glucose-first strategy may be due to bioenergetic stress induced by accumulated lactic acid. Specifically, the introduced heterologous oxidoreductase pathway (XR-XDH) faces inherent redox cofactor imbalances due to the disparate cofactor preferences of XR (NADPH) and XDH (NAD+). This redox constraint, combined with the metabolic bottlenecks of the pentose phosphate pathway (PPP), imposes a substantially greater metabolic burden compared to glycolysis (Jeffries, 2006). When xylose assimilation is initiated in the presence of high organic acid concentrations (low pH), the cell must divert a substantial portion of its limited ATP pool to maintain intracellular pH homeostasis via proton pumping (Ullah et al., 2013). Consequently, initiating fermentation with xylose when lactic acid concentrations are low allows the cells to establish a more robust metabolic flux through the PPP before the onset of severe organic acid stress, thereby maximizing overall carbon conversion efficiency (Suthers and Maranas, 2022). Ultimately, these results highlight that strategic strain engineering is required to ensure robust and efficient utilization of both glucose and xylose, thereby overcoming the inherent metabolic limitations for high-yield organic acid production in *I. orientalis*.

### 3.2 Systematic evaluation of hexokinase deletions for mixed-sugar fermentation

To attenuate the dominant glucose catabolism in *I. orientalis*, we first identified the genetic components responsible for the initial phosphorylation of glucose (Supplementary Figures S1, S2 and Table S4). Homology-based sequence analysis revealed four putative hexokinase genes, all of which contained the conserved PF00349 (hexokinase 1) and PF03727 (hexokinase 2) domains, confirming their identity as members of the hexokinase family. To gain deeper functional insights into these candidates, a detailed domain analysis was performed using the NCBI Conserved Domain Database. The results showed that HXK1 and HXK2 belong to cd24087, which represents the nucleotide-binding domain (NBD) of fungal hexokinase isozymes PI and PII. Meanwhile, HXK3 was explicitly mapped to cd24088, corresponding to the NBD of fungal glucokinases (GLK-1/2) (Herrero et al., 1995). These three isoforms could be the primary functional machinery highly optimized for sugar catabolism in this yeast. Conversely, HXK4 retained only the ancestral root domain, cd24000 (ASKHA_NBD_HK), and was predicted to lack the fungal-specific structural evolution required for efficient sugar utilization. Because HXK4 was predicted to have a negligible role in glucose metabolism during standard fermentation, it was excluded from the engineering targets.

**Figure 2:**
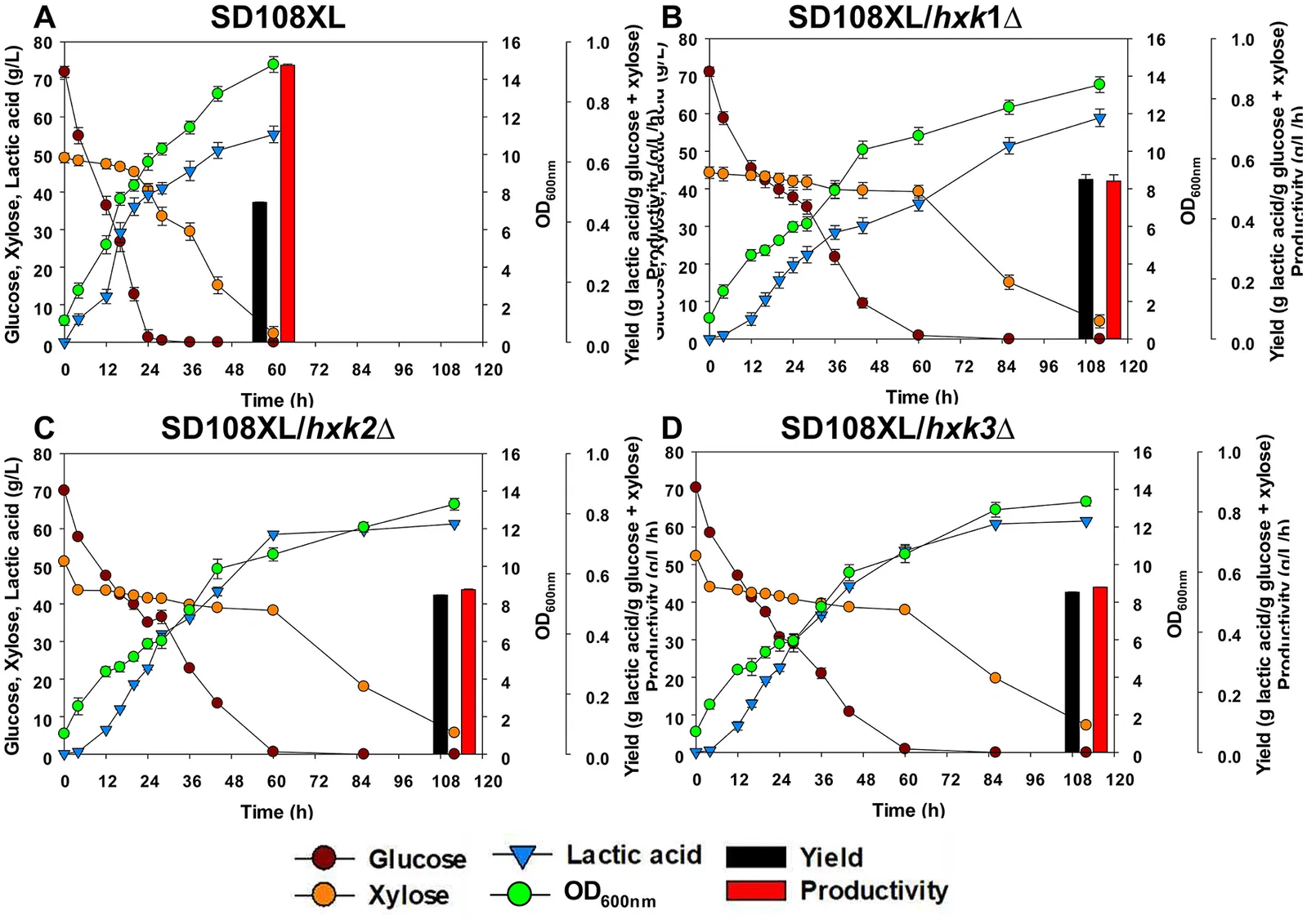
Mixed-sugar fermentation profiles of the SD108XL and its single-hexokinase-deletion mutants. Fermentation time courses are shown for **(A)** the parental strain SD108XL, **(B)** the *hxk*1Δ mutant, **(C)** the *hxk*2Δ mutant, and **(D)** the *hxk*3Δ mutant. Cells were cultivated in YP medium containing a mixture of glucose and xylose. The initial concentrations of glucose and xylose were about 70–72 g/L and 44–52 g/L, respectively. Fermentations were conducted in YP medium (25 ml in 125-ml Erlenmeyer flasks) at 30 °C with shaking at 250 rpm. To control the pH during fermentation, CaCO_3_ was added at a concentration of 50 g/L as a buffering agent. Data points and error bars represent the mean ± standard deviation from biological duplicates.

To verify the catalytic role of the identified hexokinases, the parental strain SD108XL and the single-deletion mutants (*hxk1*Δ, *hxk2*Δ, and *hxk3*Δ) were evaluated in YP medium containing glucose as the sole carbon source (Supplementary Figures S2, S3). The parental strain SD108XL exhibited rapid glucose consumption, completely depleting the substrate within 24 h. In contrast, all single-deletion mutants exhibited a distinct delay in glucose utilization, with the substrate depleted within 36 h. Despite these delays, all mutants achieved lactic acid titers comparable to those of the parental strain, confirming that although the rate of glycolysis was attenuated, the metabolic pathway for lactic acid production remained intact. When cultured in a mixed-sugar medium (approximately 70 g/L glucose and 45 g/L xylose), all single-deletion mutants also exhibited a characteristic delay in glucose utilization compared with the parental strain (Figure 2). While SD108XL depleted glucose within 28 h, the mutants required approximately 60 h to complete sugar consumption. Concurrently, xylose utilization was also delayed in all deletion strains. Whereas, the parental strain consumed almost all of the xylose within 60 h, the mutants exhibited prolonged xylose consumption profiles extending to 108–156 h. Despite the reduced consumption rates, the final lactic acid titers reached 58.9 g/L (*hxk*1Δ), 61.3 g/L (*hxk2*Δ), and 61.6 g/L (*hxk*3Δ), all of which were higher than the 55.3 g/L achieved by the parental strain (Figure 2A). This was reflected in the overall lactic acid yields, which increased from 0.47 g/g (SD108XL) to 0.53 g/g in the mutants. This suggests that the partial attenuation of glycolytic flux may have shifted the distribution of carbon away from biomass synthesis and toward the lactic acid biosynthetic pathway, as inferred from the increased product yield. However, this single deletion of hexokinase was accompanied by a reduction in volumetric productivity, decreasing from 0.922 g/L·h in the parental strain to 0.526–0.550 g/L·h in the mutants (Figure 2). This decrease in productivity was attributed to the slower sugar uptake rates caused by the attenuated glycolytic flux.

To eliminate glucose-mediated repression, we constructed a triple-deletion strain (SD108XL/*hxk123*Δ) intended to block all hexokinase-dependent glucose phosphorylation (Lane et al., 2018). We hypothesized that this would force the cells to rely exclusively on xylose metabolism, even in the presence of glucose. However, the triple-deletion mutant exhibited an unexpected and severe metabolic defect (Supplementary Figure S4). Specifically, glucose was not consumed, and the strain concurrently lost the ability to utilize xylose. We hypothesize that this total metabolic arrest could be attributed to competitive inhibition at the level of transport, resulting from a profound systemic shutdown of the native sugar uptake machinery (Ozcan and Johnston, 1999; Farwick et al., 2014). Based on established yeast regulatory models, the complete absence of early glycolytic flux and hexokinase-mediated signaling via HXK1, HXK2, and HXK3 could potentially perturb intracellular regulatory circuits, such as the Snf3/Rgt2 or Mig1 networks, that are necessary for transitioning to alternative carbon sources (Lane et al., 2018; Rolland et al., 2002; Kaniak et al., 2004). It is possible that without the requisite phosphorylation signals, the cell enters a perceived starvation state that fails to induce the expression of native hexose transporters, essentially locking the cell membrane and preventing the entry of both glucose and xylose.

### 3.3 Engineering of heterologous sugar transporters for enhanced mixed-sugar co-utilization

Although the single deletion of hexokinase genes successfully redirected carbon flux toward lactic acid production and improved overall yield (up to 0.53 g/g), it also reduced volumetric productivity due to an attenuated glycolytic rate and prolonged fermentation time. These results indicated that while lowering glucose catabolic dominance is beneficial for carbon utilization, it creates a severe kinetic bottleneck. Therefore, we sought an alternative strategy to enhance xylose assimilation without compromising the overall metabolic vigor. We focused on the transport level, the initial step of cellular sugar metabolism, by introducing heterologous sugar transporters that have been reported to function independently of glucose-mediated inhibition. Based on previous literature, *LST1* (a semi-specific transporter from *Lipomyces starkeyi*) and *AtSWEET7* (a sugar transporter from *Arabidopsis thaliana*) were selected as candidates due to their demonstrated ability to co-transport glucose and xylose simultaneously (Supplementary Figures S5, S6) (Kuanyshev et al., 2021).

To evaluate whether these transporters could bypass the total metabolic arrest observed in the glucose-phosphorylation-deficient background, *LST1* and *AtSWEET7*, driven by a strong constitutive promoter (*P*_*TDH*3_), were individually integrated into the triple-deletion strain (SD108XL/*hxk*123Δ). The SD108XL/*hxk*123Δ strain showed negligible metabolic activity even after prolonged incubation (Supplementary Figure S4). In contrast, the constitutive expression of heterologous transporters successfully re-enabled sugar influx in this background (Supplementary Figure S7). These observations suggest that heterologous transport can effectively bypass the regulatory shutdown of native transport systems. Both the *LST1*- and *AtSWEET7*-expressing triple-deletion mutant strains (SD108XL/*hxk*123Δ) initiated sugar uptake after approximately 48 h of lag phase. By 144 h, these strains achieved lactic acid titers of 9.4 g/L, and utilized all glucose (approximately 36–38 g/L) and xylose (31–40 g/L), despite reduced hexokinase activity. We speculate that the unexpected resumption of glucose metabolism in this background may be attributable to residual HXK4 activity, which was initially excluded from the deletion targets due to its ancestral, unoptimized structure. The enhanced sugar uptake and subsequent high intracellular accumulation of glucose by the heterologous transporters could potentially provide enough substrate driving force to overcome the inherently low affinity of HXK4, thereby slowly restoring basal glycolytic flux after the prolonged lag phase (Farwick et al., 2014; Li et al., 2016). This partial recovery of fermentative activity supports the hypothesis that heterologous transporters might effectively supply sugars to the intracellular metabolic pool, potentially alleviating the impact of native sensor-mediated regulatory constraints (Westholm et al., 2008; Kaniak et al., 2004). However, the overall sugar utilization rates and final titers remained markedly lower than those of the SD108XL strain, suggesting that while transport was restored, the lack of robust internal phosphorylation remained a limiting factor for high-flux production.

**Figure 3:**
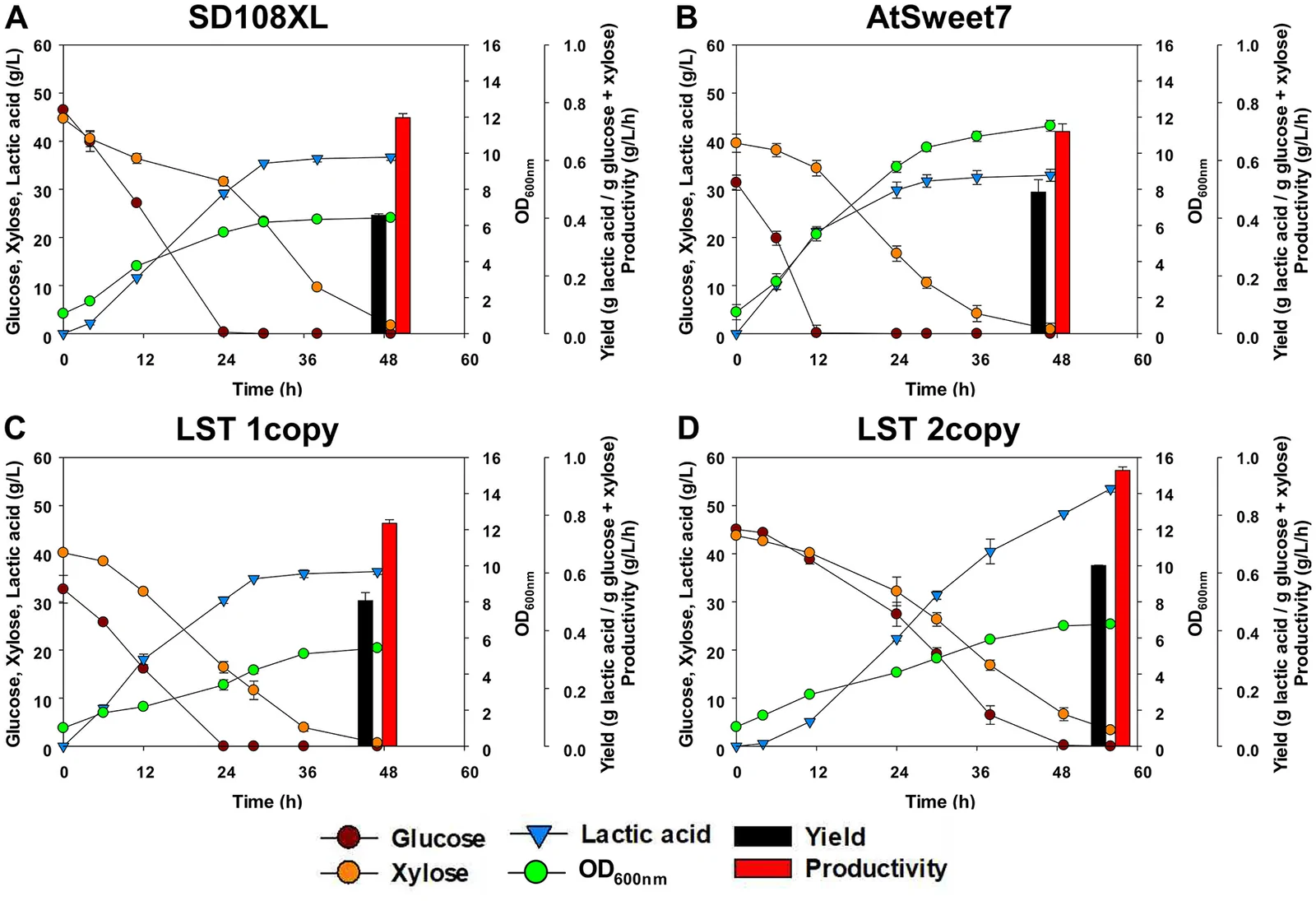
Mixed-sugar fermentation profiles of the SD108XL and its heterologous transporter-expressing strains. Fermentation time courses are shown for **(A)** the parental strain SD108XL, **(B)** the SD108XL-AS strain, **(C)** the SD108XL-LST1 strain, and **(D)** the SD108XL-LST2 strain. Cells were cultivated in YP medium containing a mixture of glucose and xylose. The initial concentrations of both glucose and xylose were added to range between 30 and 46 g/L. Fermentations were conducted in YP medium (25 ml in 125-ml Erlenmeyer flasks) at 30 °C with shaking at 250 rpm. To control the pH during fermentation, CaCO_3_ was added at a concentration of 50 g/L as a buffering agent. Data points and error bars represent the mean ± standard deviation from biological duplicates.

Thus, we shifted our strategy to introducing these transporters into the intact SD108XL background. By maintaining endogenous hexokinase activity, we aimed to decouple the transport-level limitations from the internal phosphorylation capacity. When integrated as a single genomic copy into SD108XL, the performance of the two transporters differed (Figure 3 and Supplementary Figure S6). The *AtSWEET7*-expressing strain (SD108XL-AS) exhibited sugar consumption and lactic acid production profiles nearly identical to those of the parental strain (Figures 3A, B). Specifically, SD108XL-AS maintained a distinct sequential utilization pattern, characterized by rapid glucose depletion followed by xylose metabolism. Consequently, the strain achieved a lactic acid yield of 0.49 g/g and a productivity of 0.70 g/L·h, which are slightly enhanced yield and decreased productivity compared to the parental SD108XL values (0.41 g/g yield and 0.75 g/L·h productivity). In contrast, the *LST1*-expressing strain (SD108XL-LST1) displayed an enhancement in the sugar co-utilization profile (Figure 3C). In the SD108XL-LST1 strain, xylose utilization initiated earlier and progressed more steadily, resulting in a lactic acid yield of 0.50 g/g and a productivity of 0.77 g/L·h. Compared to the parental control, these patterns indicate that LST1 expression partially alleviates xylose uptake limitations.

The better performance of *LST1* over *AtSWEET7* can be attributed to their differing transport mechanisms and evolutionary origins. While AtSWEET7 belongs to the plant-derived SWEET family, which typically functions through a distinct 7-transmembrane pore mechanism, it often exhibits relatively lower kinetic efficiency in yeast systems (Supplementary Figure S5) (Kuanyshev et al., 2021). In contrast, LST1 is a member of the Major Facilitator Superfamily (MFS) originating from the oleaginous yeast *Lipomyces starkeyi* (Supplementary Figure S5). MFS transporters operate via an alternating-access (rocker-switch) mechanism, allowing more robust substrate translocation through significant conformational changes between inward- and outward-facing states (Drew et al., 2021; Taveira et al., 2024; Yan, 2017; Drew and Boudker, 2016). This mechanism, combined with its evolutionary background, supports superior kinetic efficiency, with a lower *K*_*m*_ value for xylose than AtSWEET7 (Kuanyshev et al., 2021). This high substrate affinity appears optimized for the yeast cell membrane environment, facilitating a more rapid flux of pentose sugars. Furthermore, it is hypothesized that because LST1 is a yeast-derived transporter, its 12-transmembrane structure could be evolutionarily more compatible with the specific lipid composition and electrochemical gradient of the *I. orientalis* cell membrane compared to the plant-derived AtSWEET7 (Kuanyshev et al., 2021; Taveira et al., 2024; Ji et al., 2022). Beyond mere translocation, we propose that the expression of a native-like yeast MFS transporter, such as LST1, could potentially integrate with the endogenous sensor-mediated regulatory networks, thereby bypassing the regulatory bottlenecks typically caused by glucose dominance. This integration may not only provide a physical pathway for xylose entry but also facilitate the metabolic transition required for efficient organic acid production under mixed-sugar conditions. The engineered strains exhibited distinct macroscopic phenotypes, particularly the enhanced xylose co-consumption. Although direct molecular validation was not performed in this study, these phenotypic changes provide strong functional evidence of the successful expression and activity of these transporters in *I. orientalis*.

To further maximize xylose influx and fully leverage the potential of LST1, we integrated an additional copy of *LST1*, resulting in the SD108XL-LST2 strain (Supplementary Figure S6). This two-copy integration yielded the most improvement in mixed-sugar co-utilization (Figure 3D). Under mixed-sugar conditions (about 45 g/L glucose and 44 g/L xylose), the SD108XL-LST2 strain outperformed both the parental and single-copy strains throughout the cultivation period. Specifically, at 56 h, the SD108XL-LST2 strain achieved a lactic acid titer of 53.5 g/L with a yield of 0.63 g/g, representing an improvement over the 0.41 g/g observed for the parental SD108XL strain. Concurrently, volumetric productivity increased from 0.75 to 0.96 g/L·h. To evaluate the industrial applicability of this engineered strain, we subsequently cultivated SD108XL-LST2 in a real lignocellulosic biomass (rice straw) hydrolysate (Supplementary Figure S8). However, the enhanced co-utilization effect observed in the defined medium was largely diminished under these actual hydrolysate conditions. We hypothesize that this attenuation is likely due to the presence of various fermentation inhibitors inherent to the crude hydrolysate, which could negatively impact overall cellular fitness (Lyu et al., 2025). Therefore, further strain adaptation and bioprocess optimization will be required to fully leverage this transport-driven strategy for the direct use of lignocellulosic biomass.

These results collectively establish that sugar transport capacity is one of the bottlenecks in the mixed-sugar metabolism of *I. orientalis*. While traditional methods to alleviate carbon catabolite repression often involve deleting global regulators (e.g., components of the Snf1-Mig1 axis or hexokinases), these approaches can disrupt highly interconnected signaling networks, leading to pleiotropic effects that impair growth and overall robustness (Westergaard et al., 2007; Welkenhuysen et al., 2018; Kaniak et al., 2004). In contrast, our results suggest the possibility that strengthening the pull of xylose via heterologous transporters like *LST1* could partially counterbalance the native push of glucose dominance without disrupting essential glycolytic signaling. This transport-driven decoupling strategy provides a promising proof of concept for developing acid-tolerant yeast strains that could efficiently convert lignocellulosic sugars into high-value organic acids upon further scale-up validation.

## 4 Conclusion

This study identifies metabolic bottlenecks in mixed-sugar utilization and proposes a strategic approach toward achieving high-yield organic acid production in *I. orientalis*. Our investigation into substrate feeding strategies revealed that while operational adjustments can partially alleviate glucose dominance, native carbon catabolite repression remains a fundamental barrier to simultaneous metabolism. Although genetic attenuation of hexokinase activity improved overall lactic acid yield, likely reflecting an altered carbon distribution, it imposed a severe kinetic trade-off. The results further indicated that simultaneous disruption of HXK1, HXK2, and HXK3 caused a severe metabolic arrest under the tested conditions. The critical breakthrough was achieved by decoupling sugar transport from native regulatory constraints through the heterologous expression of the MFS-type transporter *LST1*. By increasing the LST1 gene dosage while maintaining endogenous hexokinase activity, the final strain, SD108XL-LST2, achieved superior fermentation performance, with noticeable improvements in both yield and productivity compared to the parental strain (SD108XL) under mixed-sugar conditions. These results suggest that robust mixed-sugar utilization can be achieved through a transport-driven strategy that can circumvent native metabolic hierarchies while preserving essential glycolytic signaling, providing a proof of concept for sustainable lignocellulosic biorefineries.

## Data Availability

The datasets presented in this study can be found in online repositories. The names of the repository/repositories and accession number(s) can be found in the article/Supplementary material.
