# Supplementary material for: Engineering the acid-tolerant yeast Issatchenkia orientalis for efficient lactic acid production via optimized glucose and xylose utilization
Source: Front Microbiol. 2026 Sep 11;17:1951033. doi: 10.3389/fmicb.2026.1951033 (PMC13612505; doi:10.3389/fmicb.2026.1951033)
Supplement: Supplementary file 1 [file Supplementary_file_2.docx]

Supplementary Material

**Supplementary Table S1.** Plasmids used in this study.

| Plasmid | Description | Source |
| --- | --- | --- |
| pTOP V2 | Cloning backbone vector for donor cassette construction | Enzynomics (Daejeon, South Korea) |
| pTOP V2_AS | pTOP V2 containing the P_TDH3_-AtSWEET7-T_MDH1_ expression cassette | This study |
| pTOP V2_LST | pTOP V2 containing the P_TDH3_-LST1-T_MDH1_ expression cassette | This study |
| pCast_IS2 | pCast-based gRNA expression vector targeting the IS2 locus | (Lee et al., 2024) |
| pCast_IS5 | pCast-based gRNA expression vector targeting the IS5 locus | (Lee et al., 2024) |
| pCast_HXK1 | pCast-based gRNA expression vector targeting HXK1 | This study |
| pCast_HXK2 | pCast-based gRNA expression vector targeting HXK2 | This study |
| pCast_HXK3 | pCast-based gRNA expression vector targeting HXK3 | This study |

**Supplementary Table S2.**  Synthesized genes for this study.

| Gene  (Accession No.) | Description |
| --- | --- |
| AtSWEET7  (NM_117154.4) | ATGGTTTTCGCCCATTTGAACCTTTTGAGGAAGATTGTCGGCATCATAGGCAATTTCATTGCTTTGTGTTTATTCTTATCGCCCACACCTACTTTTGTAAGAATAGTGAAAAAAAAGTCCGTTGAAGAATACTCTCCAATACCATATTTAGCTACCCTTATCAATTGCTTGGTTTGGGTACTTTACGGTTTGCCTACTGTGCACCCGGATTCTACATTAGTCATCACCATTAACGGTACTGGGATTTTAATCGAGATCGTATTTCTAACGATCTTCTTCGTATACTGCGGGCGTCAGAAACAAAGGTTAATTATATCCGCGGTTATTGCAGCAGAGACAGCCTTTATTGCAATTTTAGCTGTGCTAGTCTTAACTTTACAACATACAACCGAAAAAAGAACAATGTCAGTTGGTATTGTGTGTTGTGTTTTTAATGTTATGATGTATGCAAGTCCCTTGTCGGTCATGAAAATGGTTATCAAAACAAAATCTGTTGAGTTCATGCCATTTTGGTTGTCTGTTGCCGGTTTTCTAAACGCGGGTGTTTGGACTATTTATGCATTAATGCCTTTTGACCCATTTATGGCAATACCTAATGGAATTGGATGTCTATTTGGATTAGCTCAATTGATTCTTTACGGAGCATATTATAAGAGTACCAAGAGAATCATGGCAGAACGTGAAAATCAACCAGGCTATGTTGGTTTGTCAAGTGCTATAGCTAGAACAGGTTCAGAAAAGACGGCCAATACTAACCAGGAACCAAATAACGTCTAA |
| LST1  (XM_066942355) | ATGGGATTTTTAGGTTTCTTAAACAGATCACAAGCATCAGAACCAGTCGAAAAAGAATCTACAGCAACCACACCATCAGAAGGCAGAACGCCTCAGAGACCATTGTCTCCCGCGGTAGGAGTTCCACTTGAACAAGATTTTGAAACACAATTGTCAGCAAAGGATGCACCGTTGCTAGCTTTTATCTTAGGTGGTGTCGCATCGGTGGGCGGGTTTATGTTTGGTTATCAAACTGGGCAGATATCCGGCTTTCTAGAAATGTCCGACTTCAAAACTCGTTTTGCAGCTTGTAATAGTCAAACGGGACAATGTACTTTTTCTGCTGCAAGACAAGGTACTATTGTCGGACTATTTTCGATCGGGACTTTAATTGGTTCCTTAATTGCTGCTCCTATTGCAGATCGTATCGGAAGAAGGTTGACCATTTCTTTTTGGGCATTTTTCTTTATGATTGGTACCGTCTTGGAAATCTCCTCTTCACATGTCTGGGTTCAATTCGCTATGGGTCGTTTTGTTGGGGGATTAGGAATCGGCGCTTTGTCTGTTGTAGTTCCAATGTACCAGTCTGAAAGTACTCCTAGAATGATTAGGGGTGTGATCGTAAGTAGTTACCAGCTAATGGTTACACTAGGTATATGGCTTGCATATATGATAAATTGGGGAACAGAGTCTTTACAAGGAAGTCAATCTTGGCGTATTACAAATGGAATGTCATTTTTATGGGCTCTAGTCTTAGGTATAGCTATATTGGGTTTACCGGAGTCACCGAGGTATGCATACAGAGTTGGTCGTGAGGAAGAAGCCAGGAAGAATATGGCAAGGTTGTACAAATTATCGCCAAACCACCCCATCATAAACTTGGAGATACAGGAGATTCAAGAGAAATTGGATGCGGAAAGGGTTGGTGGCGACCATCCATGGTATGAAATCTTTACTGGACCAAGAATGTTGTATAGAACAATTTTAGGAATGGTCCTTCAAGCCGGTCAGCAGTTGACCGGAGCCAACTATTTTTTTTATTACGGTACCACAGTTTTCTCAGCCACTGGGTTGTCTAATTCATACGTTACCCAAATTATTTTAGGCTCTGTTTTTGTTGGCGGTACAATCGCTGGTTTGTGGTTAATTGAAAATTGTGGTAGAAGAAAAGCATTGATGGGTGGTGCAGCGTGGATGTTCATGTGTTTTATGGTTTTCGCGTTTGTTGGCCACTTCTCTCTTGACCATCAGAATCCTGAAGCAACACCTCAAGCCGGTGCCGCACTTGTAGTATTTTCCTGTTTATTTATTATAGCATTCGCTATGACCTGGGGTCCAATTGTGTGGGCTATTGTTGGCGAACTATATCCTGCACGTTATAGAGCCACTTGCATGGGGTTAGCCACCAGTTCCAACTGGTTATTCAATTTTTTGATAGGCTTCTTCACAACGTTCATTACTGGGGATATCGATTATCTTTACGGATTGGTGTTCGCTGGTTGTTGCTTCGCCTTATTCTTTATCGTCTATTTTTTCGTTATCGAAACTAAGGGTAGATCGCTTGAGGAAATTGACACGATGTACGTGTTACATGTGAACCCTATAAACAGTGCGAAATGGGATTCATCACAATTAATGAGGGACGGAGATGGTCTAGTTAATACTGATGATCTTTATTTAGAGGCTGGGGGTAATAGATTTGCGAAGGATGAACATTCGGCACATGAGATGATTCCAAGTCACGACGAAAATGCCCCAGGCCCCTCTCACACATCCCATGCTGATATTGAAAAGGCAGAGTCATCCGGTGCTGAGTCTCGTGATGCTTTATAA |

**Supplementary Table S3.**  Primers used in this study.

| Primer | Sequences (5'-3') | Purpose |
| --- | --- | --- |
| IOHXK1_DN_F | CGTTGCAACTGTAAATATCCTGAAGTC | Amplification of donor DNA fragments for HXK disruption |
| IOHXK1_DN_R | CTGCATTGGAGATGCTTTGTCGTT |  |
| IOHXK2_DN_F | CTGTGCAAACTTTTCTAAACCCTTG |  |
| IOHXK2_DN_R | ATTACTGTACAAGACCACTGGGGT |  |
| IOHXK3_DN_F | TCTTCTGTTTCTATCAGAAGTTAACC |  |
| IOHXK3_DN_R | ACTATGGTTTCTTTTTTTTTTTTCATTATCC |  |
| IS2_TRDN_F | AAAAATATTGAACCGTCGAAACGTCCCAAACAAGGAAATTGATTTAACCTGATCCAAAAGGGGTATGTC | Generation of expression cassettes (PTDH3-transporter-TMDH1 for genomic integration |
| IS2_TRDN_R | AAGGTGAGAATTCAAAATGTTATTTTTGATCATGTAAAGGCGTACAGGGTTATAAAGTTGTTCAGAGGAG |  |
| IS5_TRDN_F | CTTGATAAAACAAACACATTATAAAAACTGATAAACCATGAGAGTGTACCGATAAGTAAATTGATTTTAACCTGATCCAAAAGGGGT |  |
| IS5_TRDN_R | TATCGGAATCGTTGTGCCTTCCTTACTAACTTTCCTATACTACCTATTTTTGTTCAAAGTCGTACAGGGTTATAAAGTTGTTCAGAGG |  |
| pCast gHXK1_fwd | CGCAAATGACGGCAGGATTCTACT | Construction of gRNA expression vectors for CRISPR/Cas9-mediated HXK targeting |
| pCast gHXK1_rev | AAACAGTAGAATCCTGCCGTCATT |  |
| pCast gHXK2_fwd | CGCATTGTCTGTATGCTCAATTGC |  |
| pCast gHXK2_rev | AAACGCAATTGAGCATACAGACAA |  |
| pCast gHXK3_fwd | CGCAAGTTGTTTTCGTATCTAGCT |  |
| pCast gHXK3_rev | AAACAGCTAGATACGAAAACAACT |  |
| IOHXK1_CF_F | CGGCGCTTCGTGTGTTTATC | Verification of HXK deletion via diagnostic PCR |
| IOHXK1_CF_R | ACCCATGAAAATTGCGTCTGC |  |
| IOHXK2_CF_F | GGGCATTCTCATCCAAGGGT |  |
| IOHXK2_CF_R | TTTCTGGTCCATCATCGCCG |  |
| IOHXK3_CF_F2 | TGATGTCCGAAAGAGGGTGG |  |
| IOHXK3_CF_R2 | GTGTATTCGTTCGCCCGGAT |  |
| F_check_IS2 | GCGGGCGTGAAGTATATAAGACAGACAATATC | Confirmation of site-specific genomic integration at target loci (IS2 and IS5) |
| R_check_IS2 | ACTAATTCCAGTTGAGATACTCACGCACAAGA |  |
| F_check_IS5 | GGTGTGGAGTACCGATAGCCTATATGAATATT |  |
| R_check_IS5 | TGCTTTGTTTTGAAGCAAACCTCAAACCATTT |  |
| TCAT7_GA_F | CACAAACAAACACAATTACAAAAAATGGTTTTCGCCCATTTGAA | Construction of AtSWEET7 expression vectors via Gibson Assembly |
| TCAT7_GA_R | AAAGGAGGTTGTTGTGTTTCACCTTTAGACGTTATTTGGTTCCTGGTTAG |  |
| TCAT7Back_GA_F | CTAACCAGGAACCAAATAACGTCTAAAGGTGAAACACAACAACCTC |  |
| TCAT7Back_GA_R | TTCAAATGGGCGAAAACCATTTTTTGTAATTGTGTTTGTTTGTGTGTT |  |
| TCLST_GA_F | CAAACAAACACAATTACAAAAAATGGGATTTTTAGGTTTCTTAAACAGATCA | Construction of LST1 expression vectors via Gibson Assembly |
| TCLST_GA_R | AAAGGAGGTTGTTGTGTTTCACCTTTATAAAGCATCACGAGACTCAGC |  |
| TCLSTBack_GA_F | GCTGAGTCTCGTGATGCTTTATAAAGGTGAAACACAACAACCTCCT |  |
| TCLSTBack_GA_R | TGATCTGTTTAAGAAACCTAAAAATCCCATTTTTTGTAATTGTGTTTGTTTGTGTGTTTTG |  |

**Supplementary Table S4.**  Accession numbers and coding sequences of the endogenous hexokinase genes in *I orientalis*

| Gene | Accession number | Coding Sequence (5'→3') |
| --- | --- | --- |
| HXK1 | JL09_g2945 | ATGACAAACTCCAAACTAGATTTATACACTGAAAAGCTTCACAAAGACTTTCATGTCTCTACTGTTAGCTTGCACAAGATTATTGACTATTTCATCGAAGAACTGGACAGAGGTTTACAGGATGGTACCGATCCATTGGGTATTCCAATGAATGTTGCTTGGGTCATGGAATACCCAACAGGTGACGAAACGGGCGACTATCTAGCTCTGGATATGGGAGGTACCAATTTGAGGGTCGTTATTGTCCATTTGAAGGGTAAAGGTCAGATGGAAACGGATCAAGCTTTCTACAAATTACCAGACGGTATTCGTACGGTCAAGGATAGAAACGTTCTGTTTGATTTTATGGCAGAAAAACTAGATCAATTCTTGAAGGATAAACACCCTGAAGGCATTCCAGAAAACAAGGTCTTCCCATTAGGGTTTACTTTTTCTTACCCGGCAACTCAGAGCCGTATCAACTCCGGTGTCTTACAAAGATGGACTAAAGGTTTTGATATTCCAGGCGTTGAAGGTGAAGATGTTGTTCCTTTGTTAACTGAAAAGTTAAAGGAAAGAAATGTTCCAATTAAGATTGTTGCTTTGATCAACGATACCTGTGGTACTTTAGTTGCTTCTAGATATGTTGATCCTTTGACTGAAATGGGTTGTATCTTTGGTACGGGTGTTAACGGTGCTTACTACGAAAAAGTATCAAATATTACCAAATACAAAGGTAGATTCCCTAAGGATATTCCTGAGGATTCCCCAATGCTGATCAACTGTGAGTACGGTTCCTTTGACAATGCACATAAGGTTATCCCACGTACTAGATTTGATATTACTATCGACGAAACTTCCCCAAGACCTGGCCAGCAAACTTTTGAGAAAATGACGGCAGGATTCTACTTGGGTGATTTGTTAAGGTTGACACTATTGGAAGCTTACACTGAAGGTATCACCTTTGAAGATGCGCCTGCTGAATTTGTCAAGTGTCTCAATACTCCACAGTGTATTGACGCTTCCTTTCTTTCTCGTATAGAACTTGACGAGTCTGATGATTTGGTCACTGCTAAAAAGTTATTTGCAGATGAATTCAAGTATCCTAATGCAACGACGGACGAAGTTAAATTTGCCAAGGCTGCTGCCCAATTGATCAGCAACAGAGCTGCAAGATTGTCAATCTGTGGTGTTGCTGCTGTTTGTAAGAAACGTGGCTACAAGAAATGTCATATTGCAGCTGATGGCTCTGTTTACCTGAAATATCCTAACTTTGCTAGTCGTGCAGCCGAAGGCCATGCTGACGTTTTTGGTTGGGGCCACGAAATGCCACAAAGTGAACATCCTATTAGAATTGTCACTGCCTTGGATGGTTCTGGTGTTGGTGCTGCCATTATTGCTGCACTTGCTCACAAGAGGGAAGTTGCGGGCCTCTCCCTAGGTTTAGAAGATTAA |
| HXK2 | JL09_g1398 | ATGACACAAACTTTACAAGCCGCTTTTGAAAAAACAAAGTCTGAATTGACTGTTACAACTGAAAAGTTGGAAGAAATCACAAACCATTTTGTTGAAGAACTCGAAAAGGGTATTTCTCCAGCCGGTGGTAACATTCCAATGAATCCTACTTGGGTTATGGATTATCCATTAGGTTCCGAAACTGGTGAATACTTGGCAATCGATTTAGGTGGTACCAACCTTAGAGTCATCAGAGTAACATTACAAGGTGATTCCAAATTCAAATCAGTCAATGAAAAATACCCAATTCCTGTTCCGATGAGAACCGGTAATAAAGACGATCTATTTGATTTCATTGCAAAGTCTTTAGATGATTTTATTAAGCATCAGTATGGTGAGGATTACAAAGGTGAAAAAACATGGACCAAAGGTTTTGATATTCCAGGCGTTGAAGGGCATGATGTTGTTCCGATGTTACAAGCCTCCCTTGATAAATTGAATACCCCAGTTGAGGTTGTTGCCTTGATTAATGATACTACCGGTACTTTGGTCGCTTCCAAATATTGTGATACTGAGACCATCATGGGTTTGATCTTTGGTACCGGTTGTAATGGTGCTTATTATGATTTTGCAAAGAATATTCCTAAATTAGAAGGTAAGTTAGCTAGTGATATCAATGATGAAACTCCAATGGCTATTAATTGCGAATATGGTGCTTTTGATAACGATTGGAAAGTTCTGCCAAGAACCAAGTATGATATCCAAATTGACCAAGAATCTCCAAGGCCAGGTCAGCAATTCTATGAAAAAATGATTGCTGGTTATTATTTAGGTGAAGTTTTAAGATTAATTTTATTGGACTATTATTCTCAAGGACTGGTTTTCATTAATCAGGATATCAAGTCTTTGCAAGTTCCATTTGCAATGGATACCTCCTTCCCTTCAATGATTGAAGAACATGGTCCTGAATACGCTGGAAGATTATTCAAAGATACCTTCAACATTACTCTAACTAAGGAAGAAGAGAAGGTTATCTCTGATTTGTGCCAAATCATCGGTACCCGTGCTGCCAGATTGTCTGTATGCTCAATTGCTGGTATCTGTAGAAAAATGAACTACAAATCTGGACATTGTGCCGCAGATGGTTCAGTTTTTAATAGATACCCATACTTTAAGGAGCGTGCAGCTGCTGCATTGAATGAATTATTCAAGTGGAACACAGATCCAAAGGATTACCCAATTAAATTGACTCATGCAGAAGATGGTTCTGGCGTTGGTGCGGCAGTCATTGCATGTCTTACTGAAAAAAGACTGCTTGCCGGTAAGAGTGTTGGTGCTAAATTATAA |
| HXK3 | JL09_g3837 | ATGTCCCAATTAGATCAGAAGGTAAAGGAGATTGTCGAGTCGTTTGAAGTTGAGAAGGAGCTTCTCCAAAACCTTGTGACGTTATTTATCAGGTCAGCAGAATACGGCCTAACGCATTCGTCAGTGGGGAAGGCGGCAATGCCAATGATTCCAACATACGTTACGCAGATCCCAACGGGTAAGGAGAAGGGTGTCCTTTTAGCAGCCGATTTGGGCGGCACGAACTTCAGGGTATGTTCTGTTAAGTTGAACGGAGACCACACTTTTGAGTTGAAGCAGTCCAAGAACCCGATTCCCGTGGACTTGATGAGTAGCACCACCGAAGAGTTGTTTTCGTATCTAGCTGGGAAAATTGGCCAGTTTGTGGAAACCCACCATAATGATATAGGCGATAAGATGAAGCTGGGGTTCACCTTCAGTTTCCCCGTTGAGCAGACGTCGTTGAATTCGGGAACGTTGATTAGATGGACCAAGGGGTTCAACATTGAGGATACTGTCGGTAAGGACATTGTTGCCGTTTTGCAAGACCATTTAGACCGGAAAAACATTCCTGTGCACGTTGCCGCATTGGCCAACGATACCGTTGGCACCTTGTTGGCAAGATCCTATACTGGTGAAAACAAGGAAGGCTTGACCTCGATTGGATGTATCTACGGTACTGGTACAAATGGTGCCTACAATGAGAAGGTCGAAAATATCACCAAGTTGCCCAAGGAAGTTGTTGAAGAGTTGAAGTCCAAGGGAATTGACCACATGGTTATAAACACCGAGTGGGGATCCTTTGATAATAACTTGGAGCTCTTGCCCACCACCAAGTATGATGTGGAGGTCGATAAGATTTCAGCTAATCCCGGTTATCACCTGTTTGAGAAGCGAGTCTCTGGTATGTTTTTGGGAGAAATCTTGAGACATATTTTAGTGGATCTATATGAACAGGGCCTCATGTTCACCCAGTACCCTACCATCAACGAATTACCTCATAGATTGAAAACCCCTTGGTTGTTGCATTCCGAAGGCCTGTCGATTTTTGAAATCGATGATTCCACTAACTTGATTGCCACGGAAATCGAGTTGAAAAATATGTTGAGACTACCAACAACACCAGAAGAACGTAAGGCAATCCAGAAACTGACTAGAGCTATCTCCAAGAGAGCAGCACATTTGGCCGGCGTCCCAATTGCAGCATTGACTATGAAGATTGACGCATTTAAAGGCCACAATGTGGAGGTTGATGTCGGTGTTGACGGTTCAGTTGTGGAATTCTATCCTGGTTTCAGAACTATGATGAGAGACGCCATTGCAGATACCCAGATTGGCGCCAAGGGTGAAAGAAGATTGCATGTCAACCTCTCAAAGGATGGCTCCTCTGTTGGTGCTGCCCTTTGTGCATTGTCAAACAACTCATTATAA |
| HXK4 | JL09_g2451 | ATGTCCTATCTCGTTAGTCAACTCAGAACAGATACTGAAGAAGATTCCCGGGCGCCGGTGTCGGGTCTGGAAATATTAAAGGGTGGGCCTTTACTTATTTCTACAGATAACTTCTCAGATTCATCCACAGATTCACTTGCCCTGCAGAAAAATAACACAAATTCTGCTGAAGTCAAACAGGCCATAATGTCTTATCTAAAAATACATGAAATAGAGAAGAACTTTGACACCATCACAAGAGAATTCGAAAATTCCATCCATGAGTCGCTTCAGAAATCCCAAAACTGCATGTTGCCTTGCTTCAATGTTGTCTGTGATTCAAACTGTCTGCCCGACGAATACTATGCAGTGATTGATATTGGCGGTTCGACTTTACGTATTTCGGTAATAAAGTTTTTAGAAGAAAATAAGGCGGAGTGTATTATCAATAAATCATGGGTTATATTTGATGATAATAAGCATTTAGACCAACAATTCTTTGAATGGATTGCAGGTAATTTTAAATCTTTGATTGCAAACGACATGAAGGTTTTGCTGGAGAATTCTAGGGGCTCTATGAAAGTGGGTATTACTTGGAGTTTCCCCCTATTACAAATTGAGGCACCAAACAGAGCTGTTGTTTCTGATCTAGGTAAAGGATTTTCTGTATGCTTGGAATTTAAAGGTAAAGATTTGAAGGATATCTTTGAAGAATCTTTTACCAGATTTGGTCTTGACATTGAGATCTATGCAATTATAAACGATTCGGTTTCTGGGTTTGTTGCAGGGTCTTATTTGATAAACTCCAAGCTTGGATTAGTTCAGGGAACCGGTGTTAATTCTTGCTTCTTTGTTGACAAAGAGAAACTCTCCTATGGAAAGATTGGATTTCTAAAGCCCAATTTTGGAGGGGAGAAAATCCTAGTCAATGCCGAAGCTTCTTTTTTGGGGTATCACCTCTTCCCCCATATAAGTGAGGCAGACAAAGAAATGAACCCATTGTGGGACGTAATATCCGAGCAAGAATTGCCCTATCCGCACATGACAACGGGCGACTATGGTGTGTTCCAGCCTCTTGAGGTGATTACCTCGGGGAGATACATACCTGAGATAATTCGGAGGATTCTTATGAAGACCATGGACATGGAAAAGCATTCAGAAGGAGGCGCCGAATATTCCCTGTCTGCCGAATTACTCGGCGAATTGTGTTTGTCTGATGATGATGATGATGATGATGAGGTAAGAGGCAAACTGTGCACGGCTTTACAATTGGAATATATATCAAAGGATGAAATTGGAATCCTCAAGATTATAACAGAGGTGGTGATCCATCGTGCCTCAATTGTTCTTGCTTCCTACATTATTGCCATGTTGCGAGTCTCTGGCCACATCGACACCGACATTTTGACCATCTCCGTTGTTGGGTCCATGTTGCAGCATTTCCCAAGGTACAAGGAAACCGTTCTTGAAATACTGGGGAATAAAAAACATATATACAAGCTACCCAATATTACTTTTGGATTTGTCGAGGATAGCAGTATCTACGGCGCCGCCATTGCAGCATGTGCAACCCAAAGAAGTCTATGTTTATAG |

**Supplementary Figure S1.** Amino acid sequences of hexokinases in *I. orientalis*. Schematic representation of the domain structures of the identified hexokinase candidates (HXK1–HXK4). All four protein sequences harbor the two essential structural domains characteristic of the hexokinase family: the PF00349 (hexokinase 1) and PF03727 (hexokinase 2) domains.

**Supplementary Figure S2.** Verification of targeted hexokinase gene deletions. (A) SSchematic overview of the metabolic pathway engineered in *I. orientalis*, highlighting the disruption of native glucose phosphorylation (HXK). (B) Colony PCR verification of the three single-deletion mutant strains: SD108XL/*hxk1∆*, SD108XL/*hxk2∆,* and SD108XL/*hxk3∆*. The successful knockout of each target gene was confirmed by the distinctly reduced amplicon sizes in the mutant strains compared to the unmodified parental strain (WT). The expected PCR product sizes are as follows: 1024 bp for the *hxk1∆* locus versus 2026 bp for the WT; 545 bp for the *hxk2∆* locus versus 1938 bp for the WT; and 685 bp for the *hxk3∆* locus versus 2095 bp for the WT.

**Supplementary Figure S3.** Effect of individual hexokinase deletions on growth and fermentation profiles in glucose-containing medium. Strains SD108XL (A), SD108XL/*hxk1*△ (B), SD108XL/*hxk2*△ (C), and SD108XL/*hxk3*△ (D) were cultivated in YP medium containing glucose to evaluate basic growth and fermentative performance. Cultivations were performed in 125-mL Erlenmeyer flasks with a 25 mL working volume at 30°C and 250 rpm. Data points and error bars represent the mean ± standard deviation from biological duplicates.

**Supplementary Figure S4.** Fermentation profiles of the triple hexokinase deletion strain under glucose-only and glucose-xylose mixed conditions. The SD108XL/*hxk*123∆ strain was cultivated in YP medium supplemented with (A) glucose or (B) a mixture of glucose and xylose to evaluate the impact of triple hexokinase deficiency. Cultivations were performed at 30°C and 250 rpm in 125-mL Erlenmeyer flasks. Data points and error bars represent the mean ± standard deviation from biological duplicates.

**Supplementary Figure S5.** Amino acid sequences of AtSWEET7 and LST1 transporters. The structural configurations and topological boundaries are mapped onto the primary sequences. Structural labels indicate predicted transmembrane helices (TMhelix), intracellular loops (inside), and extracellular loops (outside). The green arrows represent the functional domains identified via Pfam analysis: the MtN3_slv domain (PF03083) for *AtSWEET7* and the Sugar_tr domain (PF00083) belonging to the Major Facilitator Superfamily (MFS) clan for *LST1* (bottom panel)

**Supplementary Figure S6.** Verification of heterologous sugar transporter integrations. (A) Schematic representation of the engineered mixed-sugar co-utilization pathway. The diagram illustrates the introduction of a heterologous sugar transporter (LST1 or AtSWEET7) alongside the xylose catabolic pathway to facilitate simultaneous xylose and glucose uptake for lactic acid production. (B) Colony PCR analysis confirming the genomic integration of heterologous sugar transporters in the engineered strains: SD108XL-AS, SD108XL-LST1, and SD108XL-LST2. The left panel shows the verification of single-copy integrations at the IS5 locus. The expected amplicon sizes are 763 bp for the unmodified parental locus (WT), 2446 bp for the *AtSWEET7* integration in the SD108XL-AS strain, and 3481 bp for the single-copy LST1 integration in the SD108XL-LST1 strain. The right panel displays the verification of the second LST1 integration at the IS2 locus in the SD108XL-LST2 strain, showing an expected amplicon size of 3547 bp compared to the 829 bp band of the WT locus.

**Supplementary Figure S7.** Fermentation profiles of hexokinase-deficient *I. orientalis* strains under glucose-xylose mixed conditions. Strains (A) SD108XL/*hxk*123∆, (B) SD108XL/*hxk*123∆/LST, and (C) SD108XL/*hxk*123∆/AS were cultivated in YP medium supplemented with a mixture of glucose and xylose to evaluate the restoration of sugar utilization via heterologous expression of *LST1* or *AtSWEET7*. Cultivations were performed at 30°C and 250 rpm in 125-mL Erlenmeyer flasks. Data points and error bars represent the mean ± standard deviation from biological duplicates.

**Supplementary Figure S8. Fermentation performance of the engineered *I. orientalis* strains in rice straw hydrolysate.** (A, B) Time-course profiles of cell growth (OD600nm), sugar consumption (glucose and xylose), and lactic acid production for the parental strain SD108XL (A) and the engineered SD108XL-LST2 (LST 2 copy) strain (B). The strains were cultivated in a complex YP medium supplemented with the lignocellulosic rice straw hydrolysate. (C, D) Comparison of the overall lactic acid yield (C) and volumetric productivity (D) between the SD108XL and LST 2 copy strains at specific time points (8, 14, and 20 h) during the active fermentation phase. Error bars represent the standard deviations from biological duplicates.

**References**

LEE, Y. G., KANG, N. K., KIM, C., TRAN, V. G., CAO, M., YOSHIKUNI, Y., ZHAO, H. & JIN, Y. S. 2024. Self-Buffering system for Cost-Effective production of lactic acid from glucose and xylose using Acid-Tolerant Issatchenkia orientalis. *Bioresour Technol,* 399**,** 130641.
